# Aortic stretch and recoil create wave-pumping effect: the second heart in the systemic circulation

**DOI:** 10.1098/rsif.2024.0887

**Published:** 2025-02-19

**Authors:** Arian Aghilinejad, Coskun Bilgi, Haojie Geng, Niema M. Pahlevan

**Affiliations:** ^1^ Division of Engineering and Applied Science, California Institute of Technology, Pasadena, CA, USA; ^2^ Department of Aerospace & Mechanical Engineering, University of Southern California, Los Angeles, CA, USA; ^3^ Division of Cardiovascular Medicine, Department of Medicine, University of Southern California, Los Angeles, CA, USA

**Keywords:** aorta, wave pumping, biofluid dynamics, pulsatile flow, cardiovascular system

## Abstract

Wave propagation in the heart tube is key to establishing an early pumping mechanism, as explained by impedance pump theory in zebrafish. Though initially proposed for embryonic blood circulation, the role of impedance-like behaviour in the mature cardiovascular system remains unclear. This study focuses on the understudied physiological mechanism of longitudinal displacement in the adult aorta caused by the long-axis motion of the heart. Using magnetic resonance imaging on 159 individuals, we compared aortic displacement profiles between a control group and those with heart failure, revealing a significant difference in aortic stretch between the two groups. Building on this clinical evidence, we conducted *in vitro* experiments to isolate the effects of longitudinal aortic wave pumping by eliminating the pumping action of the heart. We identified three biomechanical properties of stretch-related longitudinal wave pumping that exhibit characteristics like impedance pump: (i) a nonlinear flow–frequency relationship, (ii) bidirectional flow, and (iii) the potential for both positive and negative flow at a fixed frequency, contingent upon the aorta’s wave speed dictating the wave state. Our results demonstrate for the first time that this mechanism generates a significant flow, potentially providing a supplementary pumping mechanism for the heart.

## 1. Introduction

The adult human heart is a four-chambered, positive-displacement, piston-type pump that sends blood into the compliant aorta in a pulsatile manner. However, the pumping heart in the early embryonic stage is a simple tube that operates based on the principles of wave dynamics [1–3]. In this simple heart (pump), there are active cells that generate haemodynamic waves by contracting the tube’s wall and passive cells that facilitate wave propagation and reflections along the tube [4–6]. Using *in vivo* embryonic zebrafish models, Forouhar *et al*. [5] demonstrated that, at this early embryonic stage, wave propagations and reflections are responsible for creating the pumping effect in the heart tube, which circulates blood throughout the body. The potential coexistence of these two pumping mechanisms following the development of the four-chambered heart has long been neglected.

This wave-based pumping phenomenon, also known as an impedance pump, is a simple valveless pumping mechanism [7–9]. In its simplest form, it requires only a single wave actuator (e.g. pinching an elastic tube) and wave reflection sites. These elements can generate a net bidirectional flow, averaged over one periodic cycle, depending on the state of wave dynamics [8,10–16]. For instance, Jung & Peskin [17] have described the flow field and waves travelling along a tube using an immersed boundary method, highlighting a strong dependence of the net flow on the frequency of excitation. The existence of the wave-pumping mechanism after the formation of four-chambered heart was first suggested by Liebau [18]. A more recent *in vitro* study [19,20], conducted in physiologically accurate models of the human aorta, suggested that the haemodynamic waves created by pulsatile flow entering the aorta can create favourable or unfavourable pumping effects, depending on the state of the waves in the aorta. However, one important aspect that has not been investigated with regard to the wave-pumping effect (similar to that of the embryonic heart) is related to the mechanical coupling between the left ventricle (LV) of the heart and the aorta and their dynamics during the contracting and relaxation phases of the heart. This study was designed to investigate whether aortic stretch (occurring during systolic contraction) and its subsequent relaxation during the diastolic phase of the heart can create a pumping effect similar to that during the embryonic stage, thereby assisting the four-chamber heart in circulating blood in the systemic vasculature.

Under normal physiological conditions, interactions between the LV and the aorta are optimized to guarantee the delivery of cardiac output with modest pulsatile haemodynamic load on the heart [21–25]. Clinicians have recently begun to recognize the significance of aortic stretch and recoil during the phases of heart contraction and relaxation [26]. However, this long-delayed attention has been focused only on the contribution of aortic stretch and recoil to the LV diastolic filling [26,27], and the full aspects of this dynamic mode have yet to be explored. Our hypothesis is that the stretched elastic aorta stores energy during heart contraction, which is then released as waves when the aortic recoils during heart relaxation, creating a wave-based pumping effect. This longitudinal pumping may provide a supplementary pumping mechanism to the heart that helps reduce its workload and can have a significant impact on individuals who suffer from cardiac dysfunction such as heart failure [14,28].

To test our hypothesis, we first examined the longitudinal displacement (stretching and recoil) of the aortic root in a clinical cohort of 159 individuals. Using magnetic resonance imaging (MRI), we extracted and compared the displacement profiles of the aortic root between control group individuals and a group of heart failure patients. In the context of this study, it is important to distinguish longitudinal displacement from cross-sectional distension. The former refers to the axial motion of the aorta along its length, whereas the latter refers to radial expansion and contraction of a small portion of the aortic wall. This study specifically focuses on the longitudinal displacement. Subsequently, recognizing the inherent difficulties in studying the isolated effects of aortic wave pumping in clinical or pre-clinical settings, we employed a physiologically accurate *in vitro* model of the human systemic circulation. This model allowed us to isolate the effects of longitudinal aortic wave pumping by eliminating the contribution of the beating four-chamber heart. Consequently, all observed flow and pumping effects could be attributed solely to aortic longitudinal wave dynamics, independent of cardiac pumping. We then fully characterized the pumping behaviour of the aortic stretch and recoil as a function of wave characteristics.

## 2. Material and methods

### 2.1. Clinical study data and analysis

A total of 159 volunteers ranging in age from 20 to 92 years old participated in our clinical study by undergoing cardiac MRI. The characteristics of the clinical sample are presented in table 1. The protocol of this study was approved by the Quorum Review Institutional Review Board and registered at clinicaltrials.gov (NCT02240979). Informed consent was obtained from each participant prior to the MRI scans. In order to demonstrate the generalizability of the concept, we recruited a heterogeneous cohort that includes heart failure with reduced ejection fraction. The non-heart failure subjects enrolled in the study through online advertisements or flyers distributed at local institutions. All MRI images were acquired with a clinical 1.5 Tesla General Electric scanner (Signa HDx v. 16) using a four-element phased array (posterior and anterior) body coil and a four-lead electrocardiogram. The volunteers were scanned with an MRI protocol that consists of a localizer, followed by Fast Imaging Employing Steady State Acquisition (FIESTA) in the sagittal, long axis, short axis and radial planes. All scans were performed without any contrast agent by breath holding at the expiration. The FIESTA sequences were performed with the scan parameters of echo time of 1 ms; repetition time of 3 ms; flip angle of 60°; acquisition matrix of 512 × 512; voxel resolution of 0.78 × 0.78 mm^2^; slice thickness of 8 mm to generate the cine images of the volunteers’ hearts at the two, three and four-chamber views at 20 frames per cardiac cycle. The longitudinal displacement of the volunteer’s aorta was measured using the two, three and four-chamber view images. The Digital Imaging and Communications in Medicine (DICOM) images were opened using the open-source Segment Research software v. 4.0 R11044c (http://segment.heiberg.se) [29]. The longitudinal motion of the aorta was quantified using the basal-apical atrioventricular plane displacement (AVPD) of the LV. The AVPD was measured by manually placing six time-resolved points at the LV base (two points for each long-axis view) at the end-diastole time frame. These points are tracked to obtain the LV longitudinal displacement throughout the cardiac cycle by the AVPD algorithm of Segment software [30]. In our analysis, we assume that the longitudinal motion at the top of the aortic arch (junction to the common carotid arteries) is minimal, and the LV apex is fixed, as shown in previous clinical studies [21,26]. Under these conditions, the longitudinal shortening of the LV base during systole (e.g. AVPD) can be considered as the aorta’s stretching amount due to their mechanical and inseparable connection at the aortic root.

**Table 1.** Baseline characteristics of clinical data (N=159).

	participants, *n*	sex, W/M	age, years	height, cm	weight, kg	body mass index, kg m^−2^
control group	124	53/71	52 ± 19	172 ± 10	76 ± 16	26 ± 4
heart failure group	35	7/28	57 ± 16	171 ± 10	81 ± 21	28 ± 7

### 2.2. *In vitro* set-up and components

**Figure 1.**
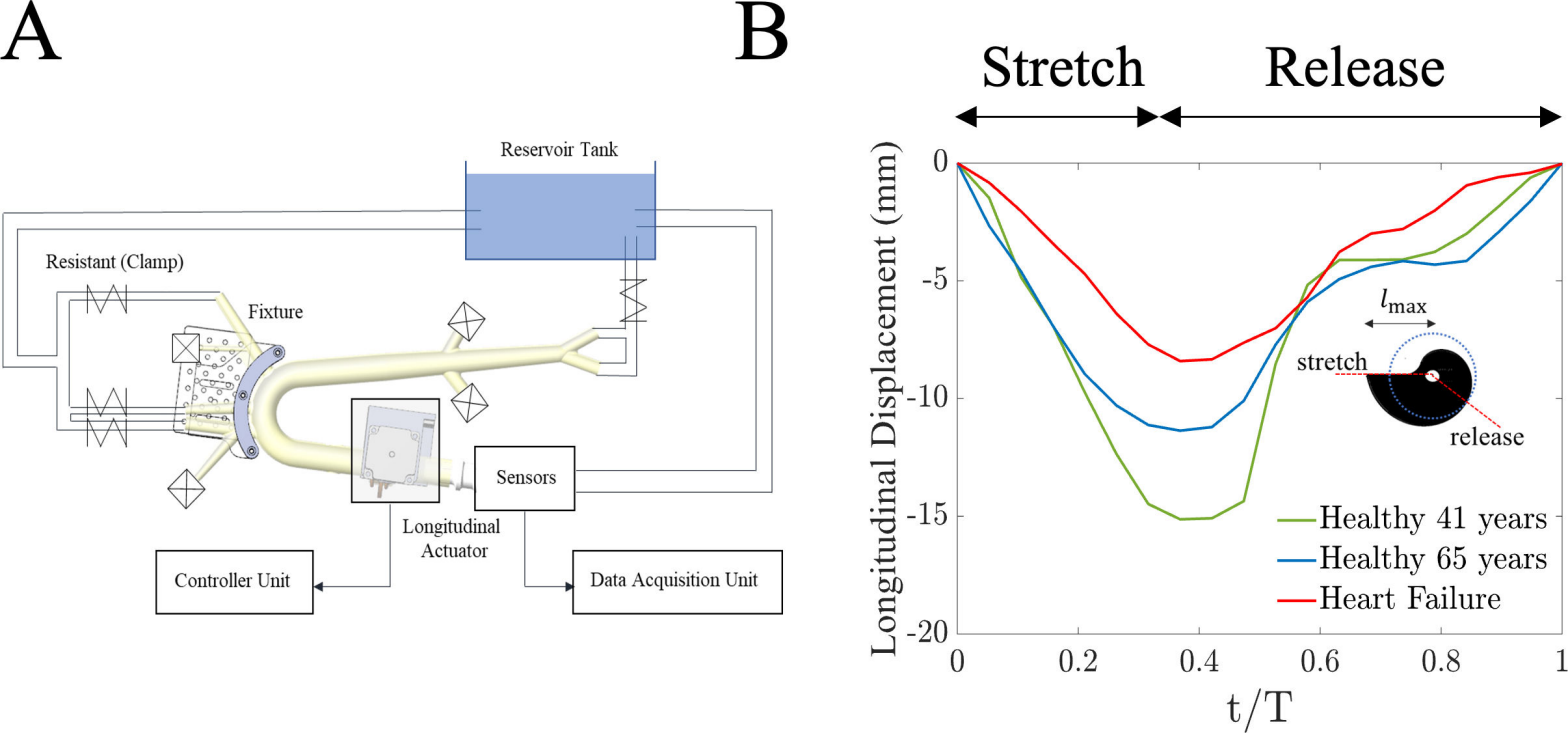
(*A*) The *in vitro* hydraulic circuit to conduct the experiments. The pressure and flow measurements are collected at the location of the sensors. (*B*) Time profile of the aortic root displacement for three cases: healthy individual of 41 years old, healthy individual of 65 years old and heart failure patient who was 43 years old. Design of the cam in the cam-follower mechanism for modelling the longitudinal stretch of the aorta is also shown in this figure.

The experimental set-up consists of a hydraulic circuit, a longitudinal stretching mechanism and an artificial human aorta. The hydraulic circuit provides a closed-loop flow circuit between the aortic phantom and the reservoir (figure 1A). In this system, the aortic phantom is installed into the system by placing luer lock-enabled barbed connectors to its major branches and connecting them with soft Tygon tubes that can be fitted to the ports on the side walls of this container. The aortic root is directly connected to the longitudinal stretching mechanism, and it is connected to the reservoir tank with a Tygon tube to complete the closed-loop circulation. Then, four resistance clamps are placed downstream of the arteries to mimic the resistances of eliminated vasculature and generate physiologically accurate flow conditions [19,20,31–34]. Natural latex and silicone-based aortas are fabricated using dip-coating and brush-coating methods, respectively (illustrated in electronic supplementary material, figure S1). Latex aortas involve dipping a mould in liquid latex, curing each layer at room temperature (25°C) for 2 h, and repeating the process as needed to achieve the desired compliance. Silicone aortas are fabricated by mixing silicone rubber (RTV-3040; Freeman Manufacturing) with a 10 : 1 base-to-catalyst ratio, degassing the mixture in a vacuum chamber, brushing it onto a silicone sheet, curing for 16 h at 25°C and repeating as necessary. Both of these materials conform with the mechanical properties of the human aorta to fabricate physiologically accurate vascular phantoms [20,31,32]. These artificial aortas are fabricated using an anatomically accurate one-to-one human-scale stainless steel mould that contains major arterial branches and aortic tapering, and all six phantoms are fabricated using the same mould (electronic supplementary material, figure S2). The characteristics of the fabricated phantom models are presented in table 2. The chosen compliance range is based on previous cardiovascular studies [35–37]. These values fall within the general reported range for human aorta and are not specific to the clinical sample in this study.

**Table 2.** Properties of the fabricated artificial aortic phantom model (N=6).

artificial aortas	wave speed, *c* (m s^−1^)	**aortic compliance** (ml mm Hg^−1^)	char. impedance (MPa s m^−3^)	material
phantom model 1	7.2	1.5	10.1	latex
phantom model 2	10.3	1.2	14.5	latex
phantom model 3	14.8	0.8	20.8	latex
phantom model 4	22.7	0.3	31.9	latex
phantom model 5	10.0	0.5	14.0	silicone
phantom model 6	15.5	0.4	21.8	silicone

The aortic phantom model is connected to the longitudinal stretching mechanism from its aortic root and the carotid arteries. The latter is placed in a fixture that constrains the movement of the aortic arch, the same as real human physiology [27]. The aortic root is connected to the stretching apparatus, which can move horizontally to generate longitudinal waves in the phantom. The actuation frequency is regulated by the software of the motor controller, whereas the maximum cam radius determines the aortic stretching amount. These cams are designed with a spiral shape that brings the stretcher to the maximum radius and holds it for 1/3 of the working period for the active stretching phase. Then, the cam releases the stretching apparatus to its initial position to create the recoil effect. Figure 1B presents three sample AVPD profiles: a 41-year-old healthy individual (green curve), a 65-year-old healthy individual (blue curve) and a heart failure patient (red curve). These cases highlight that age or disease conditions do not alter the overall displacement time profiles. The cam design is motivated by the motion of the aortic root during the systolic and diastolic phases, replicating the aorta’s longitudinal motion by stretching it at its root (e.g. AVPD). This *in vitro* set-up isolates the effects of longitudinal wave pumping in the aorta, independent of ventricle-provided ejections. The longitudinal stretch is applied to the aortic root as a controlled input using a cam-follower mechanism of varying sizes (illustrated in figure 1B), imposing a predefined stretch that initiates wave propagation and generates net flow, with flow and pressure measured as the output quantities.

A high-fidelity piezoelectric pressure catheter (Mikro-Cath, Millar Inc., Houston, TX) is inserted into the Tygon tube that connects the longitudinal stretching mechanism to the reservoir. A clamp-on ultrasonic flowmeter (ME-16PXN; Transonic Systems Inc.) is placed adjacent to the pressure sensor to avoid interference, as the ultrasonic waves from the flowmeter can generate noise that may affect the pressure sensor readings. These two sensors are connected to a PowerLab data acquisition device (ADInstruments, Colorado Springs, CO), and their data are sampled at 1 kHz frequency by LabChart Pro 7 software. For flowmeter calibration prior to experiment, a range of flow rate values (5–400 ml min^−1^) in both forward and backwards directions is introduced by a syringe pump (PHD Ultra, Harvard Apparatus, MA), and the voltage values of the flowmeter are collected. This procedure allows us to create a linear regression line for the measured voltage values against the known flow rates and obtain the calibration coefficients for this tube. The pressure catheter is calibrated to approximately 0 mmHg at the atmospheric pressure at the tube’s elevation before it is inserted into the data collection position.

### 2.3. Experimental procedure

The hydraulic circuit is filled with water, and the visible air bubbles are removed from the system. As the first step of each experiment, the data for the pulse wave velocity (PWV) analysis are collected for the phantoms. For this procedure, one pressure catheter is placed between the coronary arteries, and a second catheter is inserted into the abdominal aorta from one of the renal arteries. Consecutively, the distance between the sensor locations of these catheters is measured to compute PWV [38]. The procedure for the aortic compliance (AC) measurements can be summarized in the following steps: (i) the branches of the aortic phantoms are connected to barbed-ended connectors with luer locks (Qosina, Long Island, NY); (ii) one-way stopcock valves are attached to the luer ports; (iii) the aortic root is sealed with a connector and a plastic stopper; (iv) the phantom is filled with water, and all the air bubbles are removed through the valves; (v) a pressure catheter is inserted to the aortic phantom, and the excess water is drained until the catheter reports the atmospheric pressure (approx. 0 mm Hg); (vi) a known amount of water (Δ*V*) is injected from one of the valves; (vii) the pressure value is recorded after the catheter reports a steady value; (viii) the water injection and recording of pressure steps (vi and vii) are repeated until the phantom pressure reaches 150 mm Hg; (ix) the AC can be computed by the following formulation: $\;AC=\frac{\Delta V}{\Delta P}$; and (x) the reported AC values represent the mean compliance corresponding to the level of pressure change during the experimental procedure.

After the PWV and compliance data are collected, the stepper motor starts to actuate the stretching mechanism at 0.5 Hz until the system reaches the oscillatory steady state (10 cycles). Following this, the operating speed of the stretcher is increased by 0.5 Hz, and the system is brought to a steady oscillatory state (by waiting for 10 cycles). The previous step is repeated until the stretching mechanism is at 2.5 Hz. After the frequency range (0.5−2.5 Hz) is covered, the cam is changed to test the effect of the stretching amplitude (i.e. the cam radius), and the data collection is started at 0.5 Hz by repeating the same procedure. For the flow visualization studies, a dye solution is prepared by diluting a food colouring with tap water with a volume ratio of 1 : 50. A small amount of this dye solution (approx. 5–10 ml) is injected into the aortic root of a silicone phantom from the coronary arteries. Silicone was used for transparent aortas to facilitate optical flow visualization, while latex provided better control over compliance and PWV due to its dip-coating fabrication method. Using both materials also allowed the study to assess the impact of different phantom material properties, demonstrating the generalizability of the findings.

### 2.4. Wave intensity analysis

Wave intensity (WI) is a well-established clinical metric to quantify energy transmission through arterial waves [39,40]. WI is defined as the power per unit cross-sectional area *A*, of an artery due to blood pressure *P* = *P*(*t*) and average cross-sectional blood flow velocity *U* = *U*(*t*) [41]. Mathematically speaking, WI is computed as the product of the change in pressure (dP) times the change in velocity (dU) during a small interval, given by dI=dP.dU. To remove the dependency of the dI to the sampling time, the derivatives of pressure and velocity are divided by the time interval (denoted as $\frac{dP}{dt}$ and $\frac{dU}{dt}$, respectively), hence the WI in the units of power per unit area per unit time [42]. WI patterns determine both the direction and intensity of arterial wave propagation at any time instance during one working cycle. For example, a dI > 0 at a fixed time during the cycle indicates that forward waves are dominant at that moment. Conversely, if dI < 0, backwards waves are dominant. Based on the WI analysis and the derivative of the measured pressure, driving forces can be divided into four types based on their characteristics; forward-running compression waves where dI > 0 and dP/dt > 0; forward-running expansion waves where dI > 0 and dP/dt < 0; backwards-running compression waves where dI < 0 and dP/dt > 0; and backwards-running expansion waves where dI < 0 and dP/dt < 0.

### 2.5. Statistical analysis

The Shapiro–Wilk test for normality has been conducted in order to check the normality of the distributions of the individuals in the control and heart failure populations. Statistical significance was defined as *p* < 0.001. We performed a Student’s *t*‐test with equal variances and a two-tailed distribution to compute the statistical difference between these two populations. All statistical analysis of the clinical data was performed using Matlab (MathWorks, 2022).

## 3. Results

### 3.1. Clinical relevancy of the aortic longitudinal motion

**Figure 2.**
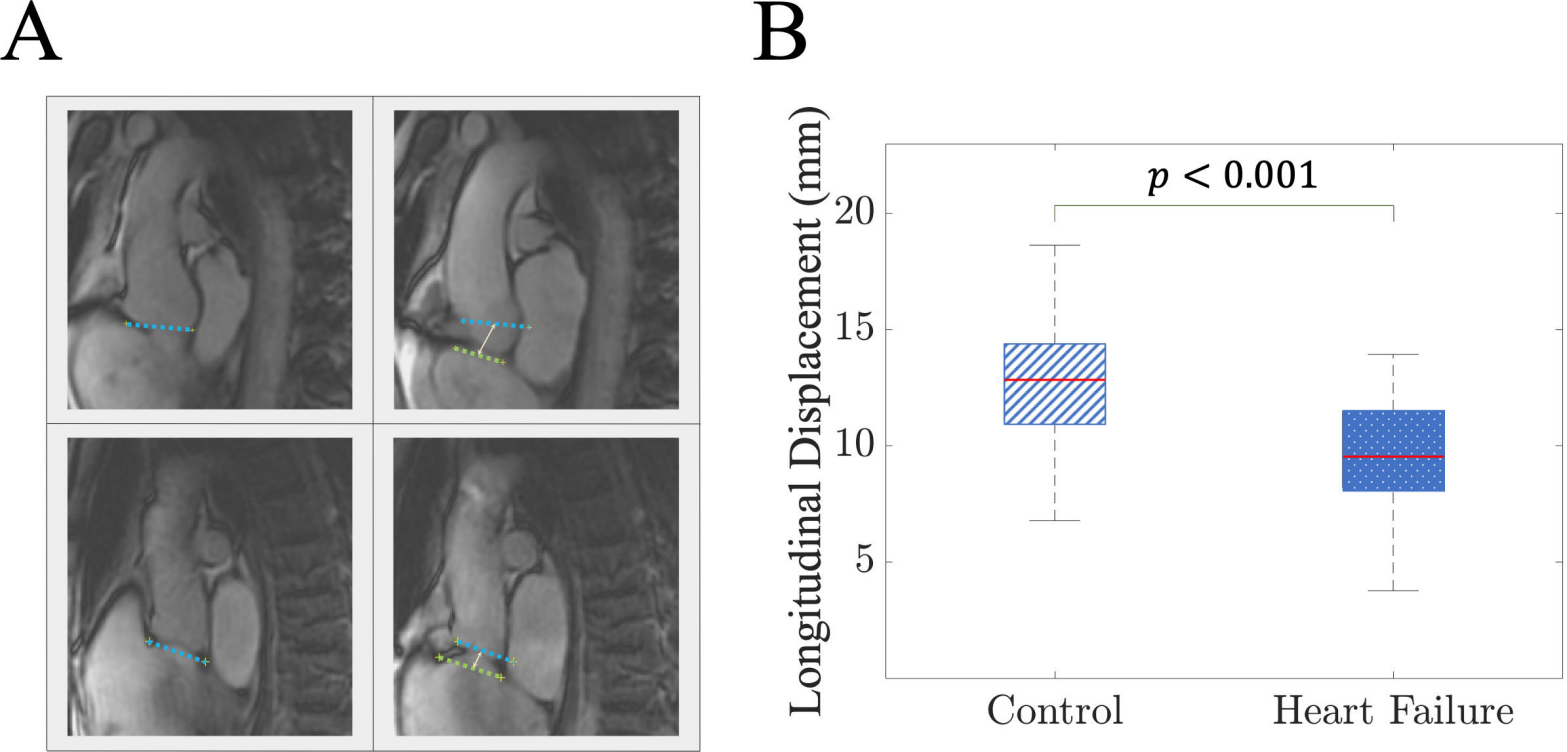
Clinical motivation and relevancy of investigating aortic wave pumping due to the longitudinal stretch. (*A*) Two pairs of magnetic resonance images captured at the end-diastole (when the aortic elements are relaxed) and the end-systole (where the maximal downward force is applied to the aortic elements, and they are positioned at their maximal stretch) for a control individual (top row) and a heart failure patient (bottom row). (*B*) The difference in the longitudinal aortic root displacement between control population and heart failure patients. *p* values suggest significant statistical differences.

To clinically assess aortic wave pumping, we used cardiac MRI to quantify longitudinal aortic motion in 159 individuals (age 53 ± 18 years, 38% women). Figure 2A illustrates magnetic resonance images of the aorta in both relaxed and stretched states for a control individual and a heart failure patient. At end-diastole, the heart muscle relaxes, and the aorta is in its unstretched length. As systole begins, due to minimal displacement of the aortic arch during systole, movement of the aortic annulus towards the apex induces longitudinal stretch of the elastic elements of the ascending aorta. At end-systole, the LV exerts maximal downward force on the aortic root, achieving maximal longitudinal stretch. In early diastole, relaxation of the LV allows the pre-loaded elastic elements in the walls of the aortic root to recoil. This recoil is passive and results from the elasticity in the aortic wall. The clinical sample (35 heart failure patients and 124 control individuals) displayed a normal distribution of longitudinal stretch (5–20 mm; electronic supplementary material, figure S3). The longitudinal aortic root displacement time profile is also provided in figure 1B for three cases: a 41-year-old healthy individual (green curve), a 65-year-old healthy individual (blue curve) and a heart failure patient (red curve). Results in figure 2B show that healthy individuals exhibit significantly higher maximal displacement than heart failure patients (*t*‐test, *p* < 0.001), indicating impaired aortic stretch and recoil as an assistive pumping mechanism in heart failure.

### 3.2. *In vitro* flow experimentation

**Figure 3.**
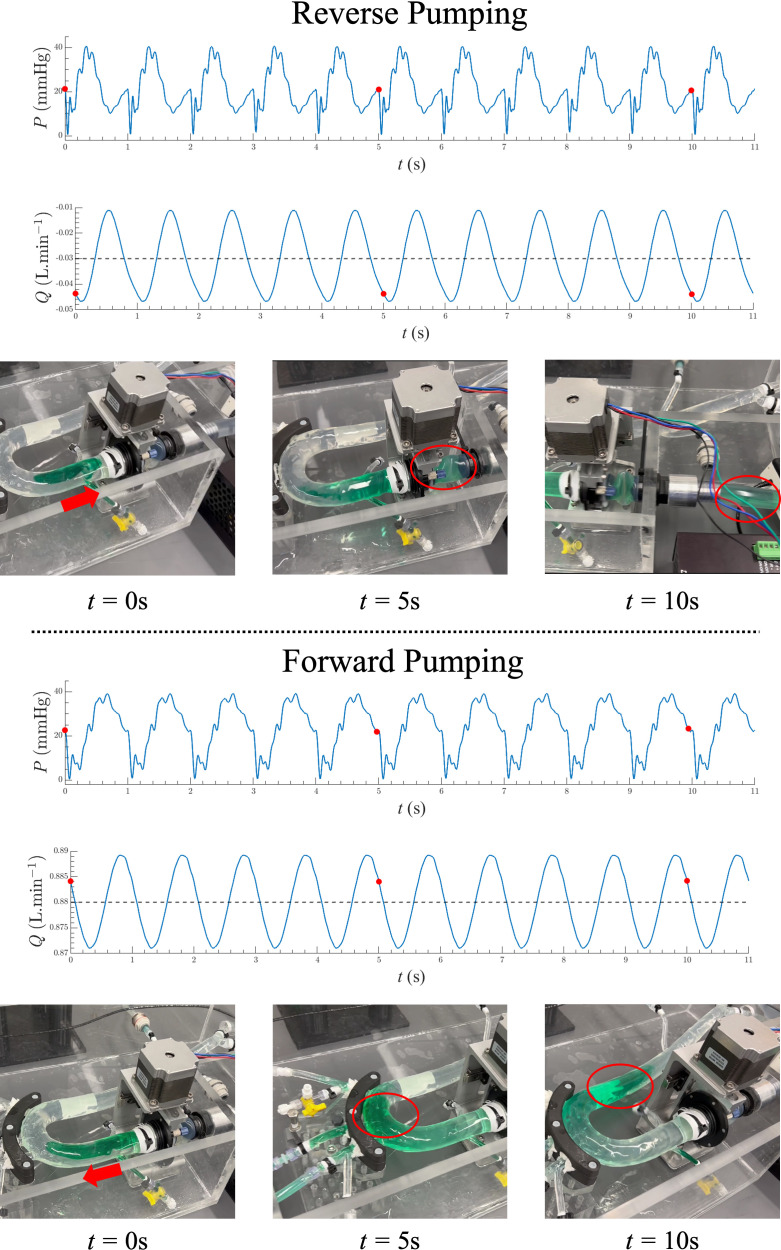
*In vitro* flow modelling and sample measurements of the aortic longitudinal wave pumping. Figures on the top panel demonstrate the generated flow direction due to the aortic stretch and recoil, excited at the frequency of 1.0 Hz at various snapshots in time that leads to reverse pumping. The installed silicone aorta is the phantom model 5. The figures on the bottom panel present the flow direction for the same aorta excited at the frequency of 1.5 Hz, leading to the forward pumping. The installed silicone aorta is the phantom model 5. Sample pressure and flow time profiles are also demonstrated at both frequencies.

Figure 3 depicts the flow direction inside the mounted aortic phantom model (model 5) with a PWV of 10 m s^−1^ at two different frequencies: 1 Hz, leading to reverse pumping (negative flow measured by the flowmeter), and 1.5 Hz, leading to forward pumping (positive flow measured by the flowmeter). Electronic supplementary material includes two sample videos corresponding to these two cases (electronic supplementary material, videos S1 and S2). These videos feature the same phantom, with alterations in stretching frequency revealing both positive and negative directions in the net-generated flow. The time–aortic displacement profile and the range of maximal displacements inspired a mechanism comprising a cam-follower design mounted on a stepper motor to actuate the aorta in the hydraulic simulator. Figure 3 also presents sample pressure and flow time profiles generated solely by aortic longitudinal stretch and recoil. The results demonstrate that net flow can be generated in both directions depending on the stretching frequency, with longitudinal wave pumping. For the same aorta, at certain frequencies, the net-generated flow is positive due to forward wave pumping, while at other frequencies, it is negative due to reverse wave pumping. Samples of similar flow waveforms and their corresponding time derivatives for measured pressure and flow waveforms for another phantom model are presented in the electronic supplementary material, figure S4, at stretching frequencies of 0.5, 1.0, 1.25, 1.5 and 2.0 Hz.

### 3.3. Effect of frequency on the aortic longitudinal wave pumping

**Figure 4.**
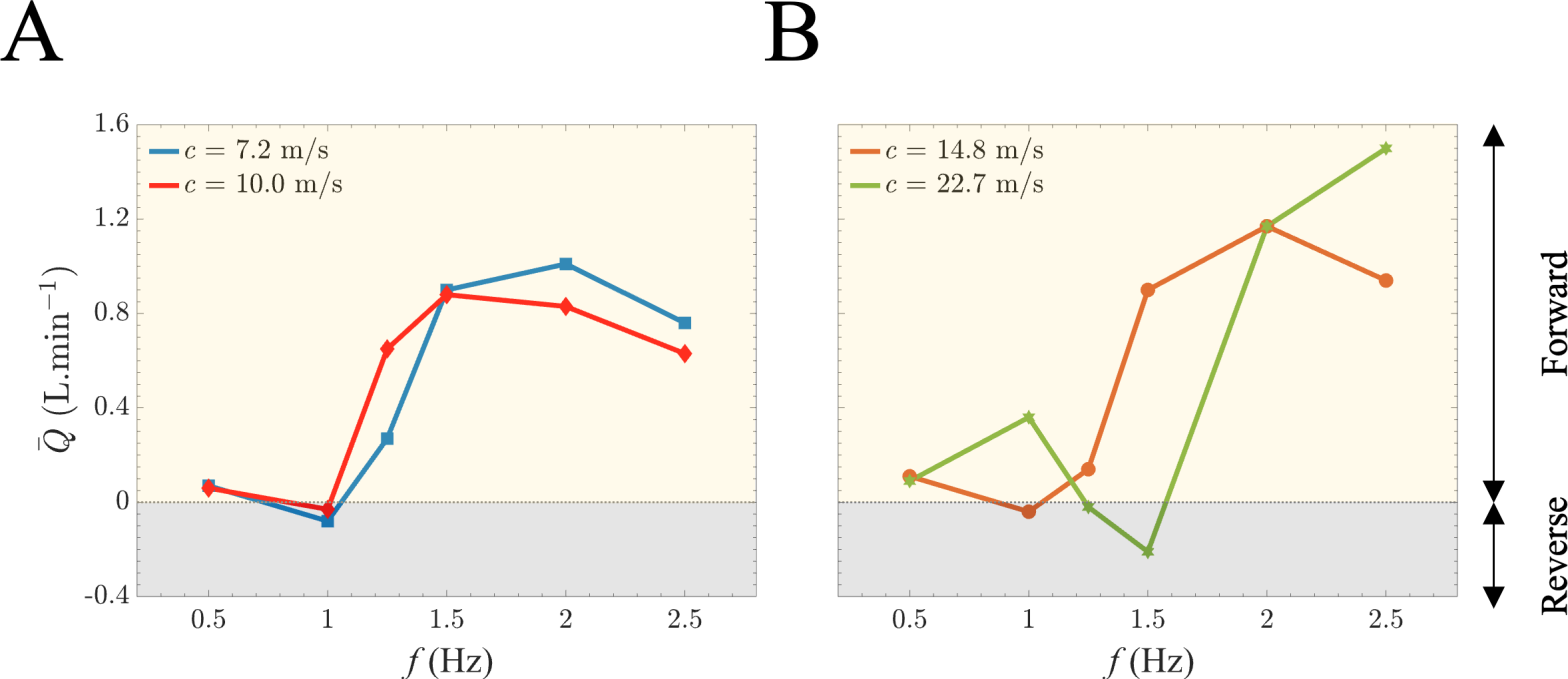
Mean flow–frequency relationship due to the aortic longitudinal stretch and recoil. (*A*) Measured mean flow rates of the aortic wave pumping against the excitation frequency for low levels of PWV (elastic region) of 7.2 and 10.0 m s^−1^. (*B*) Measured mean flow rates of the aortic wave pumping against the excitation frequency for low levels of PWV (rigid region) of 14.8 and 22.7 m s^−1^. The bidirectionality of the flow as a function of frequency exists for both cases.

Figure 4 presents the mean generated flow, $\overset{-}{Q}$, (averaged over one period T) in relation to the stretching frequency for varying aortic stiffness, as quantified by PWV. In figure 4A, the results illustrate the outcomes for two sample aortas characterized by smaller values of wave speed (phantom models 1, 5), while figure 3B demonstrates the results for aortas with larger wave speed (phantom models 3, 4). The generated flow undergoes significant, nonlinear changes as the frequency increases. This transition between modes arises from alterations in the wave state, a consequence of wave propagation and reflection. The findings indicate that the net flow can be generated in both directions depending on the stretching frequency, exhibiting longitudinal wave pumping. For a given aorta, at certain frequencies, the net-generated flow is positive due to forward wave pumping, while at other frequencies, it is negative due to reverse wave pumping. The pronounced dependency of the generated flow profile on the applied frequency is attributed to the superposition of propagating waves resulting from longitudinal stretch and reflected waves at bifurcations. Results in these figures additionally highlight a shift from forward to reverse pumping modes and vice versa, depending on the wave speed within the aorta.

### 3.4. Effect of aortic stiffness on longitudinal wave pumping

**Figure 5.**
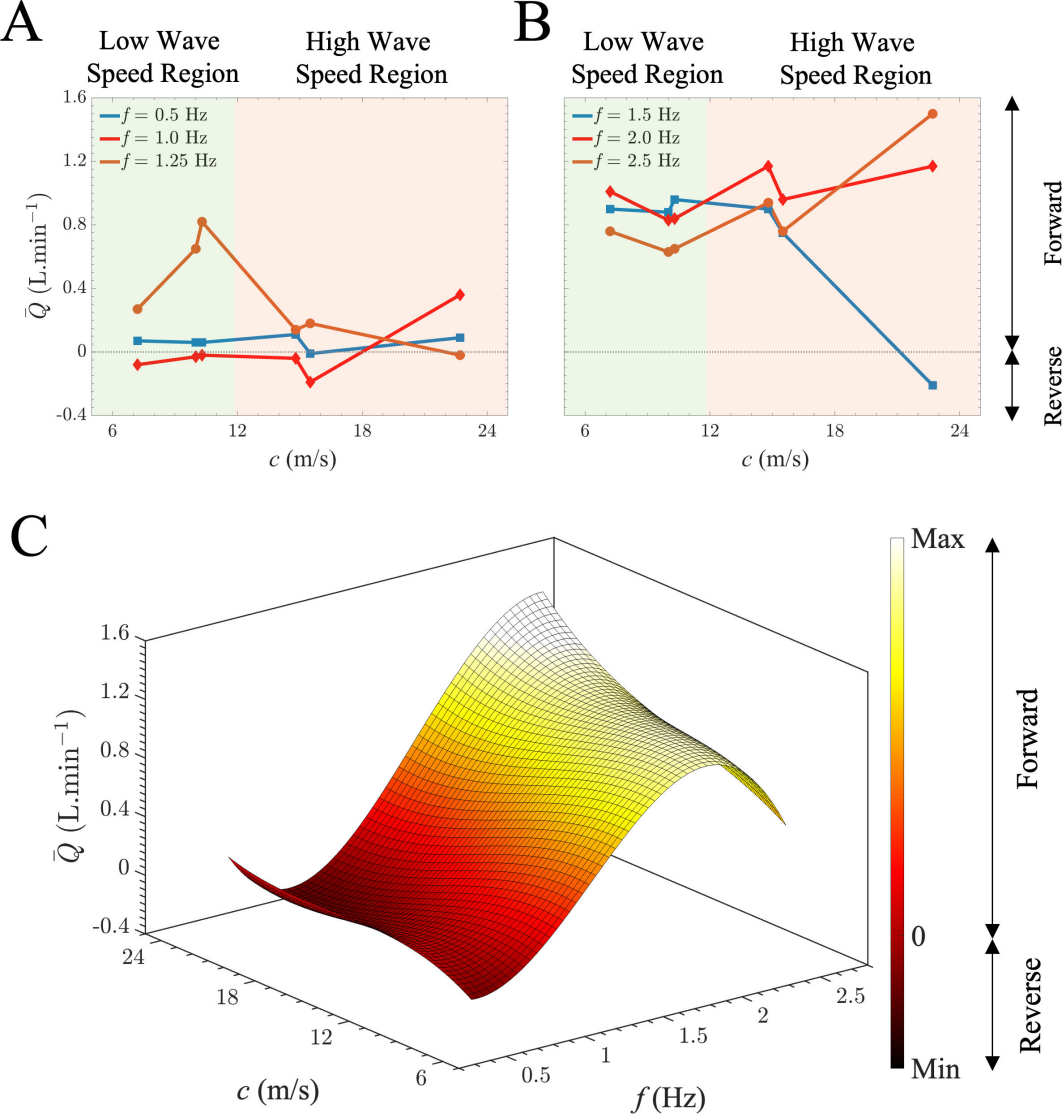
The effect of aortic stiffness, quantified by wave speed, and frequency on mean generated flow due to aortic longitudinal wave pumping. (*A*) Measured mean flow rates of the aortic wave pumping against the wave speed inside the aorta for low levels of frequency. The elastic and rigid regions are categorized based on the wave speed value. (*B*) Measured mean flow rates of the aortic wave pumping against the wave speed inside the aorta for high levels of frequency. (*C*) Interpolation mapping of the mean generated flow, $\overset{-}{Q}$, with respect to the aortic stiffness and the stretching frequency. The trend shows nonlinearity in terms of both stiffness and frequency (independent variables).

Figure 5A,*B* demonstrates the mean generated flow, $\overset{-}{Q}$, in relation to the wave speed within the aorta for various stretching frequencies. Experiments are run for six different levels of wave speeds, starting from 7.2 m s^−1^ up to 22.7 m s^−1^. In figure 5A, the results illustrate the outcomes for the aortas characterized by smaller values of frequencies (slower excitation), while figure 5B demonstrates the results for aortas stretched at larger frequencies (faster excitation). Wave speed of 12 m s^−1^ is chosen as a threshold for the low wave speed region (elastic) and high wave speed region (stiff) aortas. Results suggest that changing the wall characteristics strongly affects the net flow generation in longitudinal impedance pumps. Figure 5C depicts a three-dimensional interpolation mapping of the mean generated flow, $\overset{-}{Q}$, with respect to the aortic stiffness (as measured by wave speed) and the stretching frequency. The residuals between the measured and model-derived values from the polynomial fit (order 3) as a function of the stretching frequency and the wave speed inside the aortic phantom models are presented in electronic supplementary material, figure S5. Electronic supplementary material, table S1 shows the coefficients and their corresponding confidence interval of the model.

### 3.5. Effect of stretching amplitude on longitudinal wave pumping

**Figure 6.**
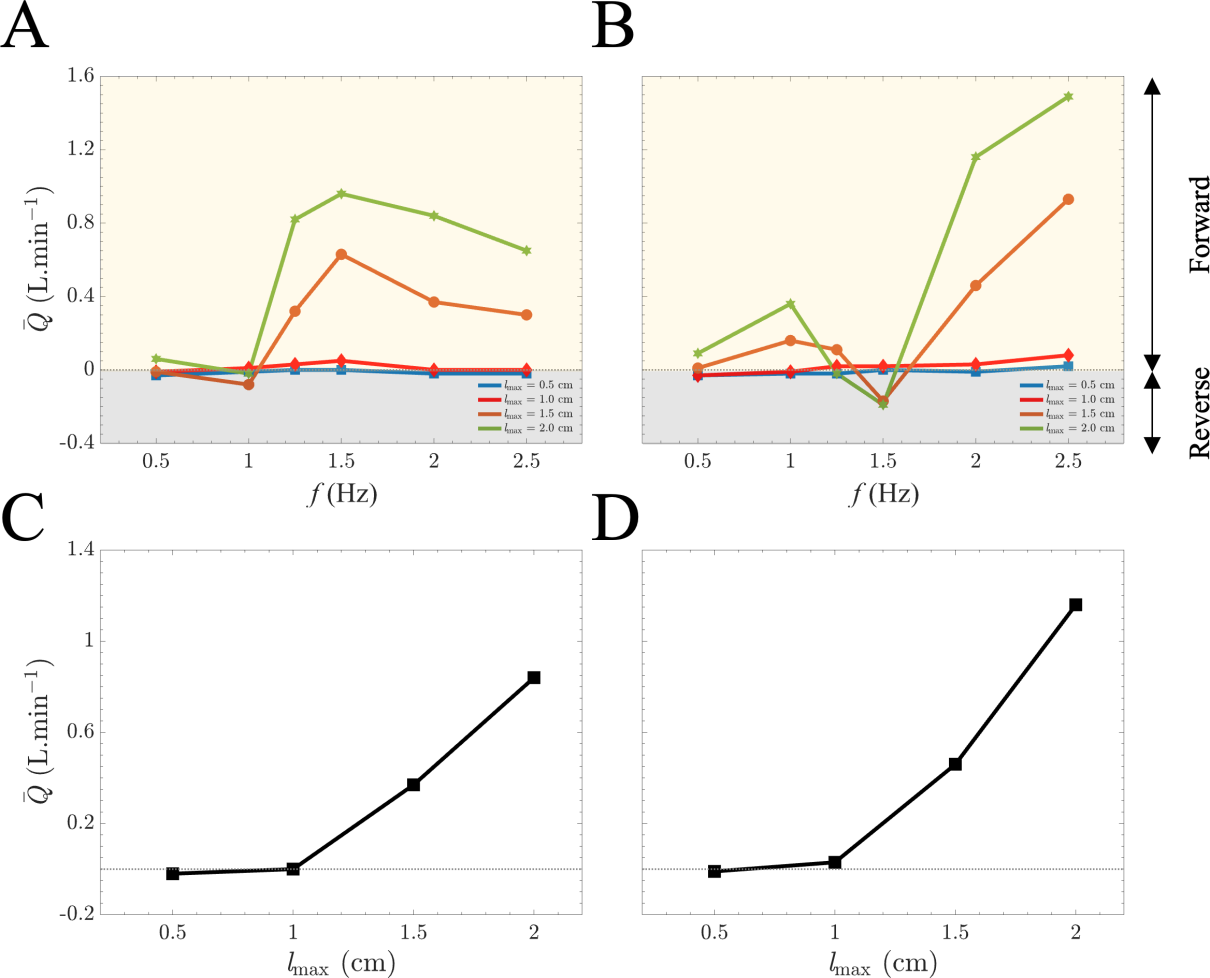
The effect of stretching amplitude on mean flow–frequency pattern due to aortic longitudinal wave pumping. Measured mean flow rates due to the aortic wave pumping against the excitation frequency for different levels of stretching amplitude of 0.5, 1.0, 1.5 and 2.0 cm for two phantom models with aortic wave speeds of (*A*) 10.3 and (*B*) 22.7 m s^−1^. For both phantom models, the flow-stretching relation follows a parabolic-like curve as demonstrated for sample case of frequency 2 Hz in (*C*) and (*D*) for wave speeds of 10.3 and 22.7 m s^−1^, respectively.

Figure 6A,B presents the mean generated flow, $\overset{-}{Q}$, as a function of frequency for different values of the longitudinal stretch, $l_{max}$, at two levels of aortic pulse wave speed of 10.3 and 22.7 m s^−1^, respectively. For each aorta, different stretching values are modelled by installing cams at different sizes. The applied stretching amplitude range is adopted based on our observations in the clinical sample, reported in electronic supplementary material, figure S3. Figure 6C,D demonstrates $\overset{-}{Q}$ as a function of stretching amplitude for each one of the aortic phantom models. Results demonstrate a nonlinear trend towards increased $\overset{-}{Q}$ as the stretching amplitude increases.

## 4. Discussion

In this study, we used a physiologically accurate *in vitro* experimental set-up and clinical cardiac MRI data to investigate a previously unnoticed wave-based mechanism in the aorta and its consequent wave-pumping effect. This aortic wave dynamics is associated with aortic stretch and recoil, caused by the LV’s long-axis shortening during its contraction and the inseparable mechanical coupling with the aortic root. Our clinical results demonstrated a significant distinction in aortic longitudinal displacement amplitude between the control group and the heart failure group. Drawing inspiration from these clinical findings, we identified three dynamical properties of this stretch-related longitudinal aortic wave pumping that are similar to those of the conventional impedance pumps: (i) a nonlinear, frequency-dependent net mean flow (averaged over the cycle); (ii) bidirectionally of the net mean flow; and (iii) dependence of both the direction and magnitude of the net mean flow on the wave speed (aortic stiffness) at a given frequency (heart rate).

### 4.1. Clinical evidence

The presence of longitudinal stretch in the ascending aorta is well-established by prior clinical observations [22,26,27]. Aortic stretch demands considerable force, with the resulting displacement representing stored energy in the elastic components of the ascending aorta. In the early phase of diastole, this stored energy from aortic stretch initiates aortic recoil, exerting an upward pull on the heart’s base [22]. Given that the heart’s apex is fixed by pericardial attachments [22,26,27], aortic recoil facilitates the lengthening of the LV in early diastole, aiding in prompt filling [26]. Overall, these previous clinical studies have demonstrated that longitudinal (axial) displacement of the aorta stores energy in spring-like elements, enhancing early diastolic recoil and contributing to the heart’s diastolic filling [27,43].

Despite these breakthrough findings, the impact of aortic longitudinal stretch and recoil on wave generation and pumping remains unexplored. Challenges in studying isolated aortic wave dynamics in *in vivo* and pre-clinical settings have hindered investigations into this matter. In this study, we evaluated aortic longitudinal stretching in 159 individuals using cardiac MRI, including 35 heart failure patients. The longitudinal displacement of the aortic root for each individual was measured as described in previous clinical studies [26,27]. Longitudinal displacement amplitude in control group and heart failure individuals was first investigated using the Shapiro–Wilk test for normality (*p* = 0.239 for control and *p* = 0.838 for heart failure). Subsequently, a Student’s *t*‐test revealed a significant difference between control and heart failure groups. This clinical evidence indicates that heart failure patients exhibit impaired mechanical coupling between the heart and the aorta, adversely affecting the longitudinal stretching of the aorta and subsequently diminishing the assistive aortic wave-pumping.

### 4.2. Physical modelling

Our results also show that longitudinal aortic wave pumping, driven by the stretch and recoil of the ascending aorta, can exhibit both positive (forward pumping) and negative (reverse pumping) flow generation, contingent on the stretching frequency (figures 3 and 4). Electronic supplementary material features dye studies for two sample videos of the same artificial aorta (electronic supplementary material, videos S1 and S2), demonstrating that both positive (forward) and negative (retrograde) net mean flow rates can be achieved in the same aortic phantom at two different frequencies (heart rates). While longitudinal wave pumping and conventional impedance pumping differ in their wave-generating systems, both mechanisms fundamentally rely on the principles of wave propagations and reflections [18,44,45]. Both the direction and magnitude of net flow vary with wall stiffness, demonstrating that alterations in wave speed affect wave travel time (figure 5) and, subsequently, interactions between propagated and reflected waves. In the context of longitudinal aortic pump flow generation, the net effect arises from stretching suction and compression waves, as has been recently shown by Aghilinejad *et al*. [14]. By modifying the stretching frequency and fixing the travel time (constant length and wave speed), coordination between the propagated and reflected waves can be achieved, sustaining net unidirectional flow. Our experimental results from figures 4 and 5 confirm this nonlinear pattern and demonstrate the strong dependency of the net-generated flow on the wave state. Our results suggest that at specific stretching frequencies (e.g. 1 Hz), varying stiffness can induce net flow in opposite directions. Furthermore, the findings in figure 5 indicate that the influence of frequency on the flow is more prominent in elastic models at lower frequencies. Conversely, this dependence is heightened in stiffer aortas (resembling those found in the elderly or individuals with diabetes) at higher frequencies. This relationship has physiological implications: in young and healthy aortas, characterized by elasticity, an increase in heart rate has a limited impact on flow generation compared with its effect on older and stiffer patients. It is noteworthy that an elevated heart rate does not automatically improve longitudinal aortic wave pumping. Instead, both heart rate and aortic stiffness must be considered together to effectively assess longitudinal aortic wave pumping, as demonstrated in figure 5.

Figure 6 illustrates the impact of stretching amplitude on the net mean flow generated by the aortic stretch and recoil mechanism. Specifically, at a frequency of 2 Hz, augmenting the stretching amplitude from 1 to 1.5 cm leads to a significant increase in net mean flow. For the aorta with a PWV of 10.3 m s^−1^, this results in a shift from no flow to 0.4 l min^−1^. Similarly, in the aorta with a pulse wave speed of 22.7 m s^−1^, the pumping effect increases from nearly no flow to 0.5 l min^−1^. This nonlinear relationship has important clinical implications, particularly given that the longitudinal stretch of the ascending aorta may substantially decrease in heart failure patients (figure 2). Our findings suggest a nonlinear parabolic relation for the flow-stretching curves that can be observed in figure 6. Notably, it has been shown previously that the energy carried by the wave is proportional to the square of the wave amplitude [19,46]. These results suggest that for maximizing the energy harvesting for the flow pumping from the aortic stretch and recoil mechanism, at a fixed frequency (heart rate), the stretching amplitude can enhance the generated net mean flow. It should be noted that while capped coronary arteries may affect flow magnitude from aortic longitudinal wave pumping, the study’s key findings—frequency dependence and nonlinear behaviour of aortic wave pumping—remain robust, driven by intrinsic wave dynamics.

#### 4.2.1. Driving mechanism in aortic wave pumping

We conducted WI analysis to better understand the wave dynamics mechanisms—a well-established method for quantifying energy within arterial waves and the net impact of forward and backwards waves [39,47,48]. Electronic supplementary material, figure S4, visuallyvisually illustrates WI patterns derived from pressure and flow measurements for a phantom stretched at different frequencies. WI patterns demonstrate that one dominant positive peak, which represents a forward-running travelling wave, is created due to the active stretching of the aortic root. This pattern is then followed by a sharp negative peak, which presents the effect of the reflected waves due to the presence of the branches, tapering and bifurcations in the aorta. These two dominant peaks are then followed by smaller positive and negative peaks that are created during the recoil of the aorta exhibiting the characteristics of a mass-spring system reacting to an initial force with an overshoot and subsequent oscillations.

**Figure 7.**
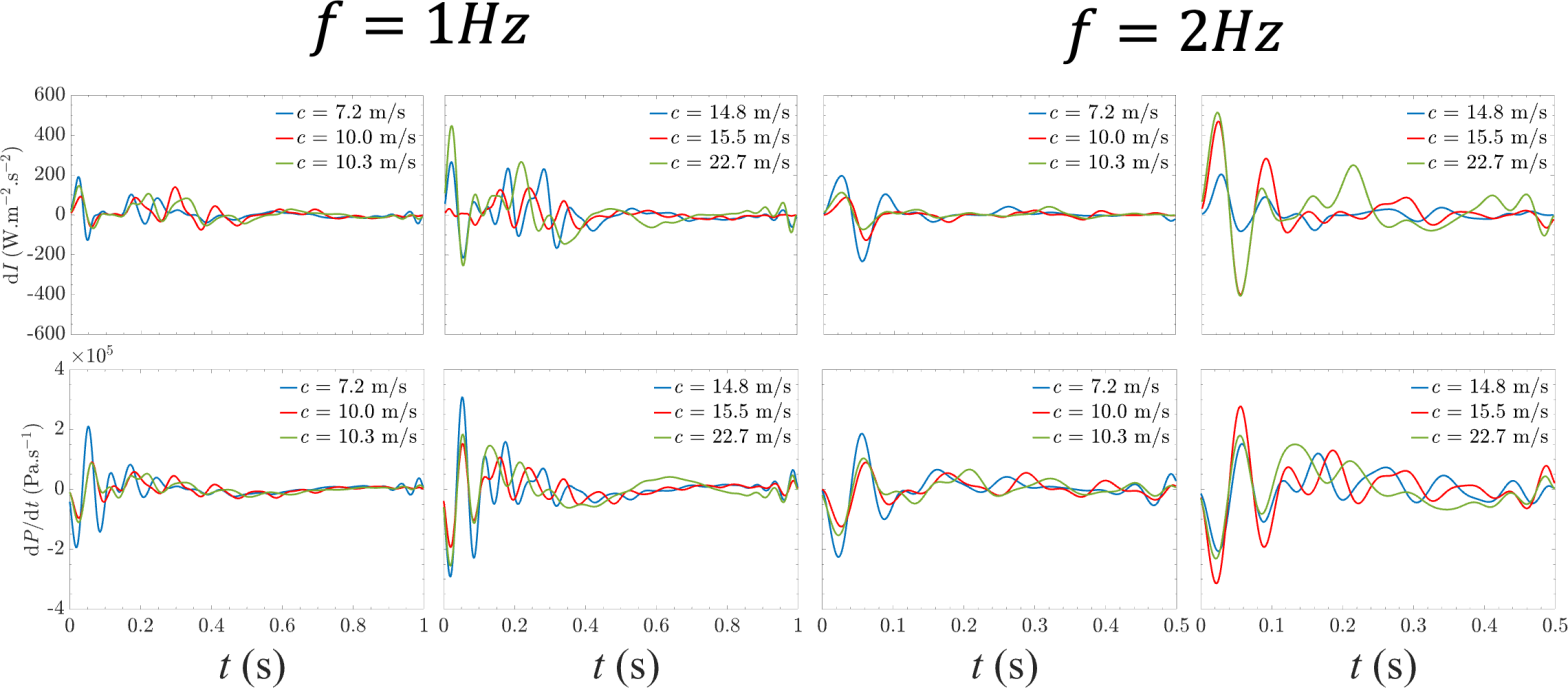
WI and time derivative of the pressure waveform patterns at different wave states. Figure demonstrates the patterns at the stretching frequency of 1 Hz for the left panel and 2 Hz for the right panel in all the phantom models with different wave speeds.

Figure 7 displays the WI patterns for aortic phantom models with varying PWV at two distinct sample frequencies. At a frequency of 1 Hz, where some aortas exhibit forward pumping and others reverse pumping, and at 2 Hz, where forward pumping prevails across all aortas, the results suggest intriguing insights. For forward pumping at 2 Hz, the WI pattern begins with forward-running waves (dI > 0). These expansion waves, resulting from aortic root stretching, propagate forward through the compliant aorta and encounter impedance mismatch at bifurcations with small arteries, leading to backwards-running compression waves. The subsequent re-reflections in the vasculature determine the evolving WI pattern. Although the initial peaks are similar between different aortas, over time, the interaction of forward and reflected waves is influenced by wave speed, causing pattern variations. At 1 Hz, the longer periodic cycle accentuates wave trapping, manifesting as low-amplitude oscillations in the WI pattern. Notably, for the aorta with a pulse wave speed of 15.5 m s^−1^, the WI pattern differs, aligning with flow measurements indicating a reverse pumping mode with net negative flow at 1 Hz (figure 5). The diminished amplitude of the initial peaks suggests that at 1 Hz, the wave state characteristics fail to generate a strong forward-running expansion wave, with the flow direction dominated by backwards-running compression waves, resulting in net negative flow. Consistent with published results in a simple straight tube with a single reflection site [14], our experimental results in this study identify two modes in the longitudinal impedance pump: reverse pumping and forward pumping. Aortic stretching induces a forward-running expansion wave, while during recoil, flow generation is primarily determined by compression reflected waves. The geometrical features of the aorta, such as the length of the ascending aorta, significantly influence aortic pumping by affecting wave dynamics. Variations in these features can alter wave propagation and reflection, impacting the superposition of forward and reflected waves.

### 4.3. Limitations and future work

This study focuses on gaining a fundamental understanding of aortic longitudinal wave pumping through *in vitro* experiments using generic phantom models. Future research should aim to explore patient-specific aspects of this phenomenon by incorporating patients’ measurements and imaging data into experimental designs. Additionally, future studies could investigate the influence of the aortic valve, including valve abnormalities, on wave-pumping dynamics. In this study, water was used as the working fluid, which differs from blood in terms of viscosity and rheological properties. Future work could address this limitation by examining the impact of viscosity and blood-like fluid properties on aortic pumping behaviour. The clinical observations highlight significant trends and suggest an impairment of the longitudinal stretching mechanism in the heart failure group. These findings warrant further comparison with clinical parameters and rigorous statistical analysis. While beyond the scope of this experimental study, such investigations represent a promising direction for future research. Lastly, this study does not account for the heterogeneous and anisotropic structure of the aortic wall, which can influence wave propagation and reflection dynamics. Additionally, variations in compliance along different sections of the aorta, from the root to the descending thoracic region [49], may impact wave speed, the timing of reflected waves and the resulting superposition of forwards- and backwards-running waves. These factors can alter the motion transfer to the fluid and affect net flow generation. However, the core findings of this study—specifically, the frequency dependence and nonlinear behaviour of aortic wave pumping—remain robust as they are intrinsic to the wave dynamics. Future work should consider incorporating spatially varying material properties and wall anisotropy to further refine the understanding of aortic wave-pumping mechanisms.

## 5. Conclusion

In conclusion, our findings indicate that aortic stretch and recoil create a pumping effect in the systemic circulation, similar to that of the conventional impedance pump. The cohort imaging data were used to establish the clinical significance of aortic root’s longitudinal motion, while the *in vitro* experiments provided a detailed physical understanding of the phenomenon. Together, these approaches complement each other, linking clinical relevance with fundamental mechanics. This pumping can generate a bimodal net flow, forward pumping mode and reverse pumping mode, depending on the state of the wave dynamics. Taking advantage of this mechanism can be of particular interest in cardiovascular diseases such as heart failure, where the pumping ability of the heart is impaired.

## Data Availability

All data are available in the main text or the supplementary materials [50].

## References

[1] Fishman MC, Chien KR. 1997 Fashioning the vertebrate heart: earliest embryonic decisions. Development **124**, 2099–2117. (10.1242/dev.124.11.2099) 9187138

[2] Liebling M, Forouhar AS, Wolleschensky R, Zimmermann B, Ankerhold R, Fraser SE, Gharib M, Dickinson ME. 2006 Rapid three‐dimensional imaging and analysis of the beating embryonic heart reveals functional changes during development. Dev. Dyn. **235**, 2940–2948. (10.1002/dvdy.20926) 16921497

[3] Forouhar A. 2004 Electrocardiographic characterization of embryonic zebrafish. In The 26th Annual Int. Conf. of the IEEE Engineering in Medicine and Biology Society (IEEE), San Francisco, CA, pp. 3615–3617. (10.1109/IEMBS.2004.1404016) 17271074

[4] Hove JR, Köster RW, Forouhar AS, Acevedo-Bolton G, Fraser SE, Gharib M. 2003 Intracardiac fluid forces are an essential epigenetic factor for embryonic cardiogenesis. Nature **421**, 172–177. (10.1038/nature01282) 12520305

[5] Forouhar AS, Liebling M, Hickerson A, Nasiraei-Moghaddam A, Tsai HJ, Hove JR, Fraser SE, Dickinson ME, Gharib M. 2006 The embryonic vertebrate heart tube is a dynamic suction pump. Science **312**, 751–753. (10.1126/science.1123775) 16675702

[6] van de Vosse FN, Stergiopulos N. 2011 Pulse wave propagation in the arterial tree. Annu. Rev. Fluid Mech. **43**, 467–499. (10.1146/annurev-fluid-122109-160730)

[7] Rinderknecht D, Hickerson AI, Gharib M. 2005 A valveless micro impedance pump driven by electromagnetic actuation. J. Micromech. Microeng. **15**, 861–866. (10.1088/0960-1317/15/4/026)

[8] Loumes L, Avrahami I, Gharib M. 2008 Resonant pumping in a multilayer impedance pump. Phys. Fluids **20**, 023103. (10.1063/1.2856528)

[9] Sarvazyan N. 2021 Building valveless impedance pumps from biological components: progress and challenges. Front. Physiol. **12**, 770906. (10.3389/fphys.2021.770906) 35173623 PMC8842681

[10] Avrahami I, Gharib M. 2008 Computational studies of resonance wave pumping in compliant tubes. J. Fluid Mech. **608**, 139–160. (10.1017/s0022112008002012)

[11] Carmigniani RA, Benoit M, Violeau D, Gharib M. 2017 Resonance wave pumping with surface waves. J. Fluid Mech. **811**, 1–36. (10.1017/jfm.2016.720)

[12] Hickerson AI, Gharib M. 2006 On the resonance of a pliant tube as a mechanism for valveless pumping. J. Fluid Mech. **555**, 141. (10.1017/s0022112006009220)

[13] Hickerson AI, Rinderknecht D, Gharib M. 2005 Experimental study of the behavior of a valveless impedance pump. Exp. Fluids 534-540 **39**, 787–787. (10.1007/s00348-005-0029-1)

[14] Aghilinejad A, Rogers B, Geng H, Pahlevan NM. 2023 On the longitudinal wave pumping in fluid-filled compliant tubes. Phys. Fluids **35**. (10.1063/5.0165150) PMC11618682 39640063

[15] Aghilinejad A, Amlani F, King KS, Pahlevan NM. 2020 Dynamic Effects of aortic arch stiffening on pulsatile energy transmission to cerebral vasculature as a determinant of brain-heart coupling. Sci. Rep. **10**, 8784. (10.1038/s41598-020-65616-7) 32472027 PMC7260194

[16] Aghilinejad A, Gharib M. 2024 Assessing pressure wave components for aortic stiffness monitoring through spectral regression learning. Eur. Heart J. Open **4**, eae040. (10.1093/ehjopen/oeae040) PMC11165314 38863521

[17] Jung E, Peskin CS. 2001 Two-dimensional simulations of valveless pumping using the immersed boundary method. SIAM J. Sci. Comput. **23**, 19–45. (10.1137/s1064827500366094)

[18] Liebau G. 1954 Über ein ventilloses pumpprinzip. Naturwissenschaften **41**, 327–327.

[19] Pahlevan NM, Gharib M. 2013 In-vitro investigation of a potential wave pumping effect in human aorta. J. Biomech. **46**, 2122–2129. (10.1016/j.jbiomech.2013.07.006) 23915578

[20] Pahlevan NM, Gharib M. 2014 A bio-inspired approach for the reduction of left ventricular workload. PLoS One **9**, e87122. (10.1371/journal.pone.0087122) 24475239 PMC3901771

[21] Bell V *et al*. 2017 Relations between aortic stiffness and left ventricular mechanical function in the community. J. Am. Heart Assoc. **6**, e004903. (10.1161/jaha.116.004903) 28069573 PMC5523643

[22] Bell V *et al*. 2015 Relations between aortic stiffness and left ventricular structure and function in older participants in the Age, Gene/Environment Susceptibility-Reykjavik Study. Circulation **8**, e003039. (10.1161/circimaging.114.003039) 25795761 PMC4380164

[23] Gharib M, Rambod E, Kheradvar A, Sahn DJ, Dabiri JO. 2006 Optimal vortex formation as an index of cardiac health. Proc. Natl Acad. Sci. USA **103**, 6305–6308. (10.1073/pnas.0600520103) 16606852 PMC1458873

[24] Aghilinejad A, Amlani F, Mazandarani SP, King KS, Pahlevan NM. 2023 Mechanistic insights on age-related changes in heart-aorta-brain hemodynamic coupling using a pulse wave model of the entire circulatory system. Am. J. Physiol. Heart Circ. Physiol. **325**, H1193–H1209. (10.1152/ajpheart.00314.2023) 37712923 PMC10908406

[25] Aghilinejad A, Wei H, Magee GA, Pahlevan NM. 2022 Model-based fluid-structure interaction approach for evaluation of thoracic endovascular aortic repair endograft length in type B aortic dissection. Front. Bioeng. Biotechnol. **10**, 825015. (10.3389/fbioe.2022.825015) 35813993 PMC9259938

[26] Bell V, Mitchell GF. 2015 Influence of vascular function and pulsatile hemodynamics on cardiac function. Curr. Hypertens. Rep. **17**, 580. (10.1007/s11906-015-0580-y) 26164466

[27] Bell V *et al*. 2014 Longitudinal and circumferential strain of the proximal aorta. J. Am. Heart Assoc. **3**, e001536. (10.1161/jaha.114.001536) 25523153 PMC4338743

[28] Pahlevan NM, Gharib M. 2014 Pathological wave dynamics: a postulate for sudden cardiac death in athletes. Med. Hypotheses **82**, 64–70. (10.1016/j.mehy.2013.11.007) 24284063

[29] Heiberg E, Sjögren J, Ugander M, Carlsson M, Engblom H, Arheden H. 2010 Design and validation of Segment - freely available software for cardiovascular image analysis. BMC Med. Imaging **10**, 1. (10.1186/1471-2342-10-1) 20064248 PMC2822815

[30] Seemann F *et al*. 2017 Time-resolved tracking of the atrioventricular plane displacement in Cardiovascular Magnetic Resonance (CMR) images. BMC Med. Imaging **17**, 16. (10.1186/s12880-017-0189-5) 28241751 PMC5330030

[31] Aghilinejad A, Wei H, Bilgi C, Paredes A, DiBartolomeo A, Magee GA, Pahlevan NM. 2023 Framework development for patient-specific compliant aortic dissection phantom model fabrication: magnetic resonance imaging validation and deep-learning segmentation. J. Biomech. Eng. **145**, 091010. (10.1115/1.4062539) 37195686

[32] Alavi R, Aghilinejad A, Wei H, Niroumandi S, Wieman S, Pahlevan NM. 2022 A coupled atrioventricular-aortic setup for in-vitro hemodynamic study of the systemic circulation: design, fabrication, and physiological relevancy. PLoS One **17**, e0267765. (10.1371/journal.pone.0267765) 36331977 PMC9635706

[33] Segers P, Dubois F, De Wachter D, Verdonck P. 1998 Role and relevancy of a cardiovascular simulator. Cardiovasc. Eng. **3**, 48–56.

[34] Timms D, Hayne M, McNeil K, Galbraith A. 2005 A complete mock circulation loop for the evaluation of left, right, and biventricular assist devices. Artif. Organs **29**, 564–572. (10.1111/j.1525-1594.2005.29094.x) 15982285

[35] Liu ZR, Ting CT, Zhu SX, Yin FC. 1989 Aortic compliance in human hypertension. Hypertension **14**, 129–136. (10.1161/01.hyp.14.2.129) 2759675

[36] Murgo JP, Westerhof N, Giolma JP, Altobelli SA. 1980 Aortic input impedance in normal man: relationship to pressure wave forms. Circulation **62**, 105–116. (10.1161/01.cir.62.1.105) 7379273

[37] Safar M, O’Rourke MF. 2006 Arterial stiffness in hypertension: handbook of hypertension series. vol. 23. Edinburgh, UK: Elsevier Health Sciences.

[38] Gaddum NR, Alastruey J, Beerbaum P, Chowienczyk P, Schaeffter T. 2013 A technical assessment of pulse wave velocity algorithms applied to non-invasive arterial waveforms. Ann. Biomed. Eng. **41**, 2617–2629. (10.1007/s10439-013-0854-y) 23817766

[39] Parker KH. 2009 An introduction to wave intensity analysis. Med. Biol. Eng. Comput. **47**, 175–188. (10.1007/s11517-009-0439-y) 19205773

[40] Parker KH, Jones C. 1990 Forward and backward running waves in the arteries: analysis using the method of characteristics. J. Biomech. Eng. **112**, 322–326. (10.1115/1.2891191) 2214715

[41] Wang JJ, Parker KH. 2004 Wave propagation in a model of the arterial circulation. J. Biomech. **37**, 457–470. (10.1016/j.jbiomech.2003.09.007) 14996557

[42] Sugawara M, Niki K, Ohte N, Okada T, Harada A. 2009 Clinical usefulness of wave intensity analysis. Med. Biol. Eng. Comput. **47**, 197–206. (10.1007/s11517-008-0388-x) 18763005

[43] Maksuti E, Bjällmark A, Broomé M. 2015 Modelling the heart with the atrioventricular plane as a piston unit. Med. Eng. Phys. **37**, 87–92. (10.1016/j.medengphy.2014.11.002) 25466260

[44] Ottesen JT. 2003 Valveless pumping in a fluid-filled closed elastic tube-system: one-dimensional theory with experimental validation. J. Math. Biol. **46**, 309–332. (10.1007/s00285-002-0179-1) 12673509

[45] Propst G. 2006 Pumping effects in models of periodically forced flow configurations. Phys. D **217**, 193–201. (10.1016/j.physd.2006.04.007)

[46] Lindsay RB. 1960 Mechanical radiation. New York, NY: McGraw-Hill Book Company.

[47] Aghilinejad A, Wei H, Pahlevan NM. 2023 Non-invasive pressure-only aortic wave intensity evaluation using hybrid Fourier decomposition-machine learning approach. IEEE Trans. Biomed. Eng. **70**, 2139–2148. (10.1109/tbme.2023.3236918) 37018682 PMC12875607

[48] Hughes AD, Park C, Ramakrishnan A, Mayet J, Chaturvedi N, Parker KH. 2020 Feasibility of estimation of aortic wave intensity using non-invasive pressure recordings in the absence of flow velocity in man. Front. Physiol. **11**, 550. (10.3389/fphys.2020.00550) 32528317 PMC7260344

[49] Krüger T, Veseli K, Lausberg H, Vöhringer L, Schneider W, Schlensak C. 2016 Regional and directional compliance of the healthy aorta: an ex vivo study in a porcine model. Interact. Cardiovasc. Thorac. Surg. **23**, 104–111. (10.1093/icvts/ivw053) 26993474 PMC4986732

[50] Aghilinejad A, Bilgi C, Geng H, Pahlevan NM. 2025 Supplementary material from: Aortic Stretch and Recoil Creates Wave Pumping Effect: the Second Heart in the Systemic Circulation. Figshare. (10.6084/m9.figshare.c.7662664)39965641

